# Dual-Subpopulation as reciprocal optional external archives for differential evolution

**DOI:** 10.1371/journal.pone.0222103

**Published:** 2019-09-19

**Authors:** Haiming Du, Zaichao Wang, Yiqun Fan, Chengjun Li, Juan Yao

**Affiliations:** 1 School of Electrical and Information Engineering, Zhengzhou University of Light Industry, Zhengzhou, Henan, China; 2 School of Computer Science, China University of Geosciences, Wuhan, Hubei, China; 3 College of Informatics, Huazhong Agricultural University, Wuhan, Hubei, China; Universidad de Granada, SPAIN

## Abstract

Differential Evolution (DE) is powerful for global optimization problems. Among DE algorithms, JADE and its variants, whose mutation strategy is DE/current-to-*p*best/1 with optional archive, have good performance. A significant feature of the above mutation strategy is that one individual for difference operation comes from the union of the optional external archive and the population. In existing DE algorithms based on the mutation strategy—JADE and its variants, individuals eliminated from the population are send to the archive. In this paper, we propose a scheme for managing the optional external archive. According to our scheme, two subpopulations are maintained in the population. Each of them regards the other as the archive. In experiments, our scheme is applied in JADE and two of its variants—SHADE and L-SHADE. Experimental results show that our scheme can enhance JADE and its variants. Moreover, it can be seen that L-SHADE with our scheme performs significantly better than four DE algorithms, CoBiDE, MPEDE, EDEV, and MLCCDE.

## Introduction

Differential evolution (DE), a type of Evolutionary Algorithm (EA) for global optimization problems, has been successfully applied in many fields [1]. In each run of DE, the population, which consists of individuals—candidate solutions of problem, need be maintained. Here, individuals are also called target vectors. In the *g*th generation of population, mutant vectors {*v*_*i*,*g*_ = (*v*_*i*,1,*g*_, *v*_*i*,2,*g*_, …, *v*_*i*,*d*,*g*_)|*i* = 1, 2, …, *NP*}, where *d* denotes dimensionality of problem, are generated through mutation based on target vectors {*x*_*i*,*g*_ = (*x*_*i*,1,*g*_, *x*_*i*,2,*g*_, …, *x*_*i*,*d*,*g*_)}. Then, crossover produces trial vectors {*u*_*i*,*g*_ = (*u*_*i*,1,*g*_, *u*_*i*,2,*g*_, …, *u*_*i*,*d*,*g*_)} based on *x*_*i*,*g*_ and *v*_*i*,*g*_. After that, *x*_*i*,*g*+1_ are selected via selection from *x*_*i*,*g*_ and *u*_*i*,*g*_ according to their fitness to problem—*f*(*x*_*i*,*g*_) and *f*(*u*_*i*,*g*_).

DE is being constantly improved at different aspects. According to [2],

- Methods based on both strategy and control parameter adaptations [3–21];
- Methods based on only strategy adaptations [1, 22–40]; and
- Methods based on population size control [41–46].

are the recent directions of DE study. Most of the above methods are explained as improving or maintaining diversity—difference among individuals. Although so many measures have been presented in literature, satisfactory solutions still cannot be obtained by DE on many occasions. Therefore, further research is still required.

JADE [3] is a state-of-the-art DE algorithm. So far, a number of variants of JADE have been proposed in literature, such as SHADE [47], *R*_*cr*_-JADE [48], L-SHADE [45], AEPD-JADE [1], JADE-SI [27], JADE_sort [20], ETI-JADE [34], and ETI-SHADE [34]. Not only JADE itself but also its variants are all based on the same mutation strategy, DE/current-to-*p*best/1 with optional archive, which is shown in Eq 1.

$$v_{i,g}=x_{i,g}+F_{i}\cdot \left(x_{best,g}^{\;p}-x_{i,g}\right)+F_{i}\cdot \left(x_{r1,g}-{\tilde{x}}_{r2,g}\right)$$ (1)

In the equation, *x*_*i*,*g*_, *x*_*r*1,*g*_ and $x_{best,g}^{\;p}$ are target vectors from population *P*. Further, $x_{best,g}^{\;p}$ is randomly chosen from the 100*p*% individuals whose fitness is better than the other individuals, where *p* ∈ (0, 1]. Meanwhile, ${\tilde{x}}_{r2,g}$ is an individual from the union of the optional external archive and the population. In addition, both *x*_*r*1,*g*_ and ${\tilde{x}}_{r2,g}$ are randomly chosen and other than *x*_*i*,*g*_.

Mutation of DE is always based on difference operation of individuals. In the majority mutation strategies, individuals for difference operation are target vectors in the current generation of population. Nevertheless, a significant feature of DE/current-to-*p*best/1 with optional archive is that one of individuals for difference operation comes from the union of the archive and the population. That is, individual for difference operation is selected from a larger range than ever. According to experimental results in literature, DE/current-to-*p*best/1 with optional archive leads to good algorithm performance.

Here, we give the motivation of this paper. In JADE and its variants, the optional external archive is managed just by a simple means. Details are given as below. In every generation, target vectors weeded out in selection are sent to archive. A sent individual is accepted by the archive only if it is different with any individuals existing in archive. That is, redundancy is not allowed in archive. When there is no free space in the archive, random individuals in it are removed for accommodating new comers. By this means, potential promising search directions in individuals eliminated from the population may be still kept for evolution. Nevertheless, under the control of the above managing method, individuals in the archive are worse in fitness than target vectors and similar in chromosome with target vectors. Hence, the method for managing the archive is not the best choice for DE/current-to-*p*best/1 with optional archive. Thus, how to manage the optional external archive need be further studied for improving DE/current-to-*p*best/1 with optional archive.

EAs naturally tend to demonstrate parallelism, since most of their variation operators can be processed in parallel. Among renowned types of parallel EAs, distributed EAs (DEAs) are most widely applied for upgrading different EAs [49]. In DEAs, the large population is divided into subpopulations for making segregation. When a predetermined condition is met, migration is executed to exchange individuals among subpopulations. By this means, for each subpopulation, foreign individuals similar in fitness level with local individuals but different in building blocks of chromosome from local individuals can be provided from time to time. Hence, upgrading an EA to a DEA can improve solutions.

Enlightened by migration of DEA, we propose a scheme to manage the optional external archive in this paper. Details are given below. The population is divided into two subpopulations. The two subpopulations evolve synchronously and independently. Each subpopulation regards the other one as its optional external archive. Between the two subpopulations, individuals are similar in fitness level with local individuals but different in building blocks of chromosome from local individuals. Therefore, under the control of our proposed scheme, individuals more fitting than before can be provided for difference operation of mutation.

Although our scheme is enlightened by migration of DEA, it differs with migration of DEA significantly. In migration, individuals from source subpopulation replace individuals in target subpopulation directly. However, under the control of our scheme, individuals from a subpopulation never migrate to the other subpopulation but just participate difference operation in mutation occurred in the latter subpopulation. In fact, DEA is very costly since multiple subpopulations need be maintained in population. However, DE with our method just need to maintain two subpopulations and then can be directly compared with existing DE algorithms.

Our experiments are based on the IEEE Congress on Evolutionary Computation 2014 (CEC2014) benchmark test suite (http://www.ntu.edu.sg/home/EPNSugan/index_files/CEC2014/CEC2014.htm). In the first of experiments, our scheme are applied in JADE and its two variants, SHADE and L-SHADE. When function dimensionality is set 30, 50 and 100, results of DE algorithms with our scheme are compared with results of the original DE algorithms. The experimental results show that our scheme can significantly improve solutions. In the second experiment, the best performer among the three DE algorithms with our method, L-SHADE with our method, is compared with four up-to-date DE algorithms—CoBiDE [6], MPEDE [12], EDEV [21], and MLCCDE [50]. The experimental results show that L-SHADE with our method is competitive in the field of DE.

The rest of this paper is organized as follows. In Section II, related work is presented. Firstly, JADE and its variants, the DE algorithms with optional external archive, are introduced. Then, DE algorithms with subpopulations are introduced. In Section III, our method for managing the optional external archive is given. Then, experimental results are shown and analyzed in Section IV. Finally, a conclusion and a prospect are dealt with in Section V.

## Related work

### JADE and its variants

JADE employs DE/current-to-*p*best with optional archive as its mutation strategy. When implementing the mutation strategy, individuals eliminated from the population are stored in the optional external archive. Moreover, in JADE, scaling factor *F* and crossover rate *CR*—the two main parameters of DE—are both adaptively set for each target vector independently. Since details of both DE/current-to-*p*best with optional archive and the existing method for managing the optional external archive has been given in the first section, we just introduce the adaptively setting of *F* and *CR* here.

As shown in Eq 2, crossover probability of each individual, which is truncated to [0, 1], is generated according to the normal distribution with mean *μ*_*CR*_ and standard deviation 0.1.

$$CR_{i}=randn_{j}\left(\mu_{CR},0.1\right)$$ (2)

If *f*(*u*_*i*,*g*_) < *f*(*x*_*i*,*g*_), the value of *CR*_*i*_ is collected into *S*_*CR*_. The mean *μ*_*CR*_ is initialized to be 0.5 and then updated after each generation according to Eq 3.

$$\mu_{CR}=(1-c)\cdot \mu_{CR}+c\cdot mean_{A}(S_{CR})$$ (3)

In Eq 3 *c* is a positive constant between 0 and 1 and *mean*_*A*_(_▪_) is the usual arithmetic mean. Similarly, as shownin Eq 4, mutation factor of each individual, which is truncated to be 1 if *F*_*i*_ ≧ 1 or regenerated if *F*_*i*_ ≦ 0, is independently generated according to Cauchy distribution with location *μ*_*F*_ and scale parameter 0.1.

$$F_{i}=randc_{i}(\mu_{F},0.1)$$ (4)

If *f*(*u*_*i*,*g*_) < *f*(*x*_*i*,*g*_), the value of *F*_*i*_ is collected into *S*_*F*_. The location parameter *μ*_*F*_ of Cauchy distribution is initialized to be 0.5 and then updated at the end of each generation according to Eq 5.

$$\mu_{F}=\left(1-c\right)\cdot \mu_{F}+c\cdot mean_{L}\left(S_{F}\right)$$ (5)

In Eq 5, *mean*_*L*_(_▪_) is Lehmer mean. According to [3], JADE outperforms jDE [51], SaDE [52], the classic DE/rand/1/bin or a canonical PSO algorithm [53].

A parameter adaptation technique which uses a historical memory of successful control parameter settings to guide the selection of future control parameter values is proposed in [47] as an enhancement to JADE. The proposed algorithm is named SHADE. According to the experimental results in [47] for the 28 CEC2013 benchmark functions, SHADE outperforms dynNP-jDE [54], SaDE, JADE, EPSDE [55] and CoDE.

A crossover rate repair technique based on successful parameters are proposed and combined with JADE in [48]. According to the technique, crossover rate is repaired by using the average number of components taken from mutant. Then, *R*_*cr*_-JADE is obtained based on the technique. The experiments results in [48] indicate that *R*_*cr*_-JADE is able to obtain significantly better solutions than JADE. Moreover, compared with jDE, SaDE, EPSDE-c [56] and CoDE, *R*_*cr*_-JADE obtains better, or at least comparable, results for the 25 CEC2005 benchmark functions.

L-SHADE, which further extends SHADE with Linear Population Size Reduction (LPSR), is proposed in [45]. LPSR continually decreases population size in runs according to a linear function. Based on the CEC2014 benchmark functions, L-SHADE is compared with dynNP-jDE, SaDE, JADE, EPSDE and CoDE as well as the state-of-the-art restart CMA-ES variants. The experimental results show that L-SHADE is quite competitive with the above evolutionary algorithms.

A mechanism, auto-enhanced population diversity, is proposed in [1]. This mechanism identifies convergence and stagnation by measuring the distribution of the population in each dimension. Once convergence is detected at a dimension, diversification is executed at that dimension. Similarly, stagnation at a dimension is eliminated as soon as it is found. The AEPD mechanism is incorporated into DE algorithms including JADE and SHADE. The results for the set of 25 CEC2005 benchmark functions show that the mechanism significantly improved the performance of JADE and SHADE. Moreover, AEPD-JADE also has a superior performance in comparison with DE/rand/1/bin [57], JADE, jDE, SaDE, CoDE, Pro DE/rand/1/bin [58], HdDE [59], and EPSDE [56], CLPSO [60] and IPOP-CMA-ES [61].

A scheme based on superior-inferior (SI) crossover is proposed in [27]. When population diversity degree is small, the SI crossover is performed to improve global search. Otherwise, the superior-superior crossover is used to enhance exploitation. The above scheme is applied in four DE algorithms including JADE. Experiments based on 24 functions selected from IEEE Swarm Intelligence Symposium 2005 and CEC2014 benchmark functions show that JADE-SI—JADE with SI crossover—is significantly better than JADE in the majority of cases.

A modified JADE version with sorting crossover rate (CR) is proposed in [20]. In the proposed algorithm JADE_sort, a smaller CR value is assigned to individual better in fitness. Based on the CEC2005 functions, JADE_sort is compared with jDE, SaDE, EPSDE, JADE, CoDE and JADE-SI. The experiments results show JADE_sort is competitive.

The event-triggered impulsive (ETI) control scheme is introduced in [34]. Two types of impulses—stabilizing impulses and destabilizing impulses, are presented. In runs, the number of individuals taking impulsive control is decided by an adaptive mechanism. After that, the decided number of individuals are chosen by ranking assignment. Then these chosen individuals are adaptively modified with the above two kinds of impulses. The ETI control scheme is incorporated into ten DE algorithms including JADE and SHADE. According to the experiments on the CEC2014 benchmark functions, ETI-JADE outperforms not only original JADE but also AEPD-JADE [1]. Also, ETI-SHADE outperforms SHADE and AEPD-SHADE [1].

### DE algorithms with subpopulations

In this subsection, we list five DE algorithms with subpopulations. The latest two of them are involved in our experiments for comparison. Although the listed DE algorithms all have more than one subpopulations, they do not belong to DEA, at least do not belong to narrow sense DEA, because different subpopulations in these algorithms are different in operators or settings. Details are given as below.

A dual-population differential evolution (DPDE) with coevolution is proposed in [62] for constrained optimization problems (COPs). In this algorithm, COPs is treated as a bi-objective optimization problem where the first objective is the actual cost or reward function to be optimized, while the second objective accounts for the degree of constraint violations. At each generation in runs, population is divided into two subpopulations based on the solution’s feasibility to treat the both objectives separately. Each subpopulation focuses on only optimizing the corresponding objective which leads to a clear division of work. Furthermore, DPDE makes use of an information-sharing strategy to exchange search information between the subpopulations.

An adaptive multiple subpopulations based DE algorithm, MPADE, is designed in [28]. In MPADE, population is split into three subpopulations based on fitness. Three DE strategies are performed on three subpopulation, respectively. Furthermore, an adaptive approach is designed for parameter adjustment in the three DE strategies. According to its replacement strategy, a few best offspring may replace worst parents.

In [24], mDE-bES is proposed. In this algorithm, population is divided into independent subpopulations, each with different mutation and update strategies. A mutation strategy that uses information from either the best individual or a randomly selected one is used. Selection of individuals for some of the tested mutation strategies utilizes fitness-based ranks of these individuals. Function evaluations are divided into epochs. At the end of each epoch, individuals are exchanged between subpopulations.

MPEDE [12] is an ensemble of multiple mutation strategies with adapted *F* and *CR*. These mutation strategies are current-to-*p*best/1, current-to-rand/1 and rand/1. Each mutation strategy controls an indicator subpopulation. After every pre-defined number of generations, the best-performing mutation strategy is found by a proposed equation. Then a reward subpopulation, which is randomly allocated to a mutation strategy at beginning, is assigned to the best-performing mutation strategy. In MPEDE, the method to adapt *F* and *CR* comes from [3].

EDEV [21] is an ensemble of differential evolution variants and consists of three state-of-the-art DE algorithms, JADE, CoDE and EPSDE. Each constituent DE variant is assigned an indicator subpopulation. According to a mechanism similar to the one in MPEDE, the most efficient constituent DE variant is determined after every pre-defined generation, Furthermore, a reward subpopulation is assigned to the currently best-performing constituent DE variant.

## Our method for managing the optional external archive

In DE, the more individuals are involved in mutation or the more individuals can be chosen for mutation, the higher mutation degree may be gotten. It can be seen from Eq 1 that, on one hand, five individuals are required in the mutation strategy. On the other hand, an individual for difference operation is chosen from a larger range than the population. Hence, compared with other mutation methods, DE/current-to-*p*best/1 with optional archive show higher mutation degree. Although the archive contains individuals as the population does, it is not another population since no new individual can be produced in it. Therefore, no function evaluation is required for maintaining the optional external archive. In brief, the archive provides additional individuals for mutation without consuming extra function evaluation or leading to high degeneration. That is, diversity of the population is improved in a reasonable manner. Hence, JADE and its variants show good performance.

In JADE or its variants, individuals in the optional external archive are ones eliminated from the population at different generations. Therefore, individuals in the optional external archive have similarities in chromosome to current target vectors since genetic relationships exist. Meanwhile, individuals in the archive are worse in fitness than target vectors because they are all losers in selection. Provided that individuals in archive are very different in chromosome with target vectors but similar in fitness level with current target vectors, DE/current-to-*p*best with optional archive may be further enhanced.

In our scheme, two subpopulations need be maintained in DE. The two subpopulations regard each other as the optional external archive. In this way, individuals in the archive are not only different in building blocks of chromosome from current target vectors, but also similar in fitness level with current target vectors. To show details of our method for managing the optional external archive, we adapt the pseudo-code of JADE. Although our method can also be used in any variants of JADE, expressing our method based on original JADE is more concise than based on one of its variant. The adapted pseudo-code is given in Algorithm 1.

**Algorithm 1** JADE With our Method for Managing the Optional External Archive

1: Set *μ*_*CR*_ = 0.5; $\mu_{CR}^{\prime }=0.5$; *μ*_*F*_ = 0.5; $\mu_{F}^{\prime }=0.5$;

2: Randomly create the initial generation of the two subpopulations *SP*_0_, {*x*_*i*,0_|*i* = 1, 2, …, *NP*/2}, and $SP_{0}^{\prime }$, $\left\{x_{i,0}^{\prime }|i=1,2,...,NP/2\right\}$

3: **for** *g* = 1 to G **do**

4:  $S_{F}=S_{F}^{\prime }=⌀$; $S_{CR}=S_{CR}^{\prime }=⌀$

5:  **for** *i* = 1 to *NP*/2 **do**

6:   Generate *CR*_*i*_ = *randn*_*i*_(*μ*_*CR*_, 0.1), $CR_{i}^{\prime }=randn_{i}\left(\mu_{CR}^{\prime },0.1\right)$, *F*_*i*_ = *randc*_*i*_(*μ*_*F*_, 0.1), $F_{i}^{\prime }=randc_{i}\left(\mu_{F}^{\prime },0.1\right)$

7:   Randomly choose ${\tilde{x}}_{r2,g}\neq x_{r1,g}\neq x_{i,g}$ from $SP_{g}\cup SP_{g}^{\prime }$ and ${\tilde{x}}_{r2^{\prime },g}^{\prime }\neq x_{r1^{\prime },g}^{\prime }\neq x_{i,g}^{\prime }$ from $SP_{g}^{\prime }\cup SP_{g}$

8:   $v_{i,g}=x_{i,g}+F_{i}\cdot \left(x_{best,g}^{p}-x_{i,g}\right)+F_{i}\cdot \left(x_{r1,g}-{\tilde{x}}_{r2,g}\right)$

9:   $v_{i,g}^{\prime }=x_{i,g}^{\prime }+F_{i}^{\prime }\cdot \left(x_{best,g}^{\prime p}-x_{i,g}^{\prime }\right)+F_{i}^{\prime }\cdot \left(x_{r1^{\prime },g}^{\prime }-{\tilde{x^{\prime }}}_{r2^{\prime },g}\right)$

10:   **for** *j* = 1 to *D* **do**

11:    **if** *j* = *j*_*rand*_ or *rand*(0, 1) < *CR*_*i*_ **then**

12:     *u*_*j*,*i*,*g*_ = *v*_*j*,*i*,*g*_

13:    **else**

14:     *u*_*j*,*i*,*g*_ = *x*_*j*,*i*,*g*_

15:    **end if**

16:    **if** $j=j_{rand}^{\prime }$ or $rand\left(0,1\right)<CR_{i}^{\prime }$ **then**

17:     $u_{j,i,g}^{\prime }=v_{j,i,g}^{\prime }$

18:    **else**

19:     $u_{j,i,g}^{\prime }=x_{j,i,g}^{\prime }$

20:    **end if**

21:   **end for**

22:   **if** *f*(*x*_*i*,*g*_) < *f*(*u*_*i*,*g*_) **then**

23:    *x*_*i*,*g*+1_ = *x*_*i*,*g*_

24:   **else**

25:    *x*_*i*,*g*+1_ = *u*_*i*,*g*_, *CR*_*i*_ → *S*_*CR*_, *F*_*i*_ → *S*_*F*_

26:   **end if**

27:   **if** $f\left(x_{i,g}^{\prime }\right)<f\left(u_{i,g}^{\prime }\right)$ **then**

28:    $x_{i,g+1}^{\prime }=x_{i,g}^{\prime }$

29:   **else**

30:    $x_{i,g+1}^{\prime }=u_{i,g}^{\prime }$, $CR_{i}^{\prime }\to S_{CR}^{\prime }$, $F_{i}^{\prime }\to S_{F}^{\prime }$

31:   **end if**

32:  **end for**

33: **end for**

## Experimental studies

Our experiments are based on the 30 CEC2014 benchmark test functions. In the first experiment, original version of JADE and its variants is compared with their version based on our scheme. Then, the best performer among DE algorithms with our scheme is compared with up-to-date DE algorithms in the second experiment.

### DE algorithms for experiments

For the first experiment, we need to select variants of JADE beside JADE itself. As mentioned above, SHADE, *R*_*cr*_-JADE, L-SHADE, AEPD-JADE, JADE-SI, JADE_sort, ETI-JADE and ETI-SHADE are variants of JADE. Among these algorithms, L-SHADE, AEPD-JADE, ETI-JADE and ETI-SHADE are tested based on the CEC 2014 benchmark functions in literature. According to results in [1, 34, 45], it can be seen that L-SHADE performs much better on the CEC2014 functions than the other algorithms. Thus, we select L-SHADE for the first experiment. In addition, SHADE, the foundation of L-SHADE and an variant of JADE, also be selected by us. In short, our method is employed in the three algorithms, JADE, SHADE and L-SHADE, for the first experiment.

For the second experiment, we chose CoBiDE, MPDED, EDEV, and MLCCDE to compare with the best performer among DE algorithms with our scheme. CoBiDE is a state-of-the-art DE algorithm having no relationship with JADE. MPDED and EDEV are recent DE algorithms with subpopulations and belong to related work. MLCCDE is one of the most recent DE algorithms.

### Settings

Function dimensionality is set 30, 50 and 100, respectively, in the first experiment, while only 30 in the second experiment. According to the guideline of CEC 2014 competition, maximum fitness evaluations are set 10000 ⋅ *D* for the all DE algorithms, where *D* represents function dimensionality. All parameters of the original DE algorithms are given in Table 1 based on [3, 6, 12, 21, 45, 47, 50], respectively. It can be seen from Table 1 that we change population size *NP* in the original algorithms to arrange two subpopulations for implement our scheme. In the DE algorithms with our method, each subpopulation is allocated *NP*/2 individuals and regard the other subpopulation as its archive.

**Table 1:** Settings.

Algorithm	Parameters
JADE	*μ*_*F*_ = 0.5, *μ*_*CR*_ = 0.5, *c* = 0.1 and *p* = 0.05, |*A*| = 100 and *NP* = 100
JADE with our method	*μ*_*F*_ = 0.5, *μ*_*CR*_ = 0.5, *c* = 0.1 and *p* = 0.05, and *NP* = 150
SHADE	*H* = 100, *NP* = 100 and |*A*| = 100
SHADE with our method	*H* = 150, and *NP* = 150
L-SHADE	*H* = 6, |*A*| = 2.6 ⋅ 18 ⋅ *D*, and *NP*_*init*_ = *round*(18 ⋅ *D*)
L-SHADE with our method	*H* = 6, and *NP*_*init*_ = *round*(1.4 ⋅ 18 ⋅ *D*)
CoBiDE	*pb* = 0.4, *ps* = 0.5, *NP* = 60
MPEDE	λ_1_ = λ_2_ = λ_3_ = 0.2, λ_4_ = 0.4, *ng* = 20, and *NP* = 250
EDEV	λ_1_ = λ_2_ = λ_3_ = 0.1, λ_4_ = 0.7, *ng* = 20, and *NP* = 60
MLCCDE	*M*_*F*_ = 0.7,*M*_*CR*_ = 0.5,*H* = 100,*T* = 300,*G*_*T*_ = 1500,*SR*_*T*_ = 0(*G* < *G*_*T*_), *SR*_*T*_ = 0.1(*G* > *G*_*T*_), *N* = 0.05, and *NP* = 100

### Comparison between DE algorithms with our scheme and their original version

Experimental results of original DE algorithms and DE algorithms with our method for functions with 30, 50 and 100 dimensions are listed in Tables 2–4, respectively. According to Table 2, when function dimensionality is 30, our method significantly improves JADE in ten cases out of 30 ones, SHADE in 9/30 cases and L-SHADE in 10/30 cases. Meanwhile, our method statistically deteriorates JADE in 4/30, SHADE in 3/30 cases and L-SHADE in 2/30 cases. In addition, there is no significant difference in other cases. According to Table 3, when function dimensionality is 50, our method significantly improves JADE in 11/30 ones, SHADE in 10/30 cases and L-SHADE in 9/30 cases. Meanwhile, our method statistically deteriorates JADE in 3/30, SHADE in 4/30 cases and L-SHADE in 3/30 cases. In addition, there is no significant difference in other cases. According to Table 4, when function dimensionality is 100, our method significantly improves JADE in 9/30 ones, SHADE in 11/30 cases and L-SHADE in 9/30 cases. Meanwhile, our method statistically deteriorates JADE, SHADE and L-SHADE in two cases, respectively. In addition, there is no significant difference in other cases.

**Table 2:** Results of DE algorithms with our scheme and original DE algorithms when function dimensionality is set 30. “+” denotes the result of a DE algorithm with our method is significant better than the result of its original DE algorithm in terms of Wilcoxon’s rank sum test at a 0.05 significance level, while “−” represents statistical worse. In addition, “≈” shows that there is no significant difference.

Function	Mean error (standard deviation)					
	JADE	JADE with our method	SHADE	SHADE with our method	L-SHADE	L-SHADE with our method
F1	3.00E+02 (4.99E+02)	1.12E+02 (3.47E+02)≈	3.72E+02 (1.09E+03)	8.43E+01 (5.83E+02)≈	0.00E+00 (0.00E+00)	0.00E+00 (0.00E+00)≈
F2	1.89E-14 (1.36E-14)	8.53E-15 (1.30E-14)≈	0.00E+00 (0.00E+00)	0.00E+00 (0.00E+00)≈	0.00E+00 (0.00E+00)	0.00E+00 (0.00E+00)≈
F3	4.76E-05 (1.48E-04)	1.53E-04 (6.46E-04)≈	0.00E+00 (0.00E+00)	0.00E+00 (0.00E+00)≈	0.00E+00 (0.00E+00)	0.00E+00 (0.00E+00)≈
F4	9.09E-14 (4.63E-14)	4.55E-14 (1.08E-13)+	0.00E+00 (0.00E+00)	0.00E+00 (0.00E+00)≈	0.00E+00 (0.00E+00)	0.00E+00 (0.00E+00)≈
F5	2.03E+01 (3.07E-02)	2.02E+01 (1.49E-02)+	2.01E+01 (2.06E-02)	2.01E+01 (2.36E-02)≈	2.01E+01 (2.15E-02)	2.01E+01 (2.24E-02)≈
F6	9.88E+00 (1.55E+00)	2.74E+00 (8.67E-01)+	3.48E-01 (6.73E-01)	2.16E-01 (3.24E-01)+	0.00E+00 (0.00E+00)	0.00E+00 (0.00E+00)≈
F7	2.47E-04 (1.35E-03)	3.79E-15 (2.45E-14)+	0.00E+00 (0.00E+00)	0.00E+00 (0.00E+00)≈	0.00E+00 (0.00E+00)	0.00E+00 (0.00E+00)≈
F8	0.00E+00 (0.00E+00)	0.00E+00 (0.00E+00)≈	0.00E+00 (0.00E+00)	0.00E+00 (0.00E+00)≈	0.00E+00 (0.00E+00)	0.00E+00 (0.00E+00)≈
F9	2.63E+01 (4.16E+00)	8.72E+00 (2.18E+00)+	1.58E+01 (4.22E+00)	1.46E+01 (2.49E+00)≈	6.88E+00 (1.53E+00)	4.21E+00 (9.72E-01)+
F10	6.94E-03 (1.26E-02)	2.78E-03 (7.20E-03)≈	5.55E-03 (1.08E-02)	2.78E-03 (9.97E-03)≈	5.55E-03 (1.08E-02)	1.39E-03 (5.28E-03)≈
F11	1.58E+03 (2.19E+02)	1.47E+03 (3.72E+02)≈	1.49E+03 (2.37E+02)	1.67E+03 (2.58E+02)−	1.20E+03 (2.02E+02)	1.15E+03 (1.16E+02)≈
F12	2.63E-01 (3.73E-02)	2.94E-01 (4.74E-02)−	1.62E-01 (2.45E-02)	1.37E-01 (2.11E-02)+	1.64E-01 (2.15E-02)	1.73E-01 (2.48E-02)−
F13	2.24E-01 (3.03E-02)	2.18E-01 (2.57E-02)≈	1.94E-01 (3.43E-02)	1.89E-01 (2.43E-02)≈	1.22E-01 (1.50E-02)	1.14E-01 (1.13E-02)+
F14	2.32E-01 (3.36E-02)	2.57E-01 (3.47E-02)−	2.46E-01 (2.98E-02)	2.31E-01 (2.32E-02)≈	2.42E-01 (3.05E-02)	2.09E-01 (1.48E-02)+
F15	3.17E+00 (4.51E-01)	3.11E+00 (3.18E-01)≈	2.50E+00 (3.77E-01)	2.28E+00 (2.75E-01)+	2.14E+00 (2.18E-01)	2.12E+00 (4.52E-01)≈
F16	9.41E+00 (3.27E-01)	9.38E+00 (2.83E-01)≈	9.09E+00 (3.65E-01)	8.46E+00 (2.34E-01)+	8.63e+00 (4.41E-01)	8.78E+00 (5.47E-01)−
F17	1.24E+03 (4.46E+02)	7.95E+02 (5.47E+02)+	1.08E+03 (3.25E+02)	8.67E+02 (1.05E+02)≈	2.01E+02 (9.71E+01)	1.49E+02 (8.41E+01)+
F18	8.04E+01 (5.84E+01)	4.19E+01 (3.61E+01)≈	5.91E+01 (2.51E+01)	2.14E+01 (9.17E+00)+	6.35E+00 (3.25E+00)	6.64E+00 (1.49E+00)≈
F19	4.38E+00 (6.06E-01)	3.76E+00 (5.24E-01)+	4.31E+00 (7.12E-01)	3.81E+00 (5.14E-01)+	3.56E+00 (5.97E-01)	3.11E+00 (6.74E-01)+
F20	3.54E+03 (2.50E+03)	8.57E+02 (1.69E+03)+	1.35E+01 (6.64E+00)	1.47E+01 (7.42E+00)−	2.99E+00 (1.18E+00)	2.25E+00 (2.74E+00)+
F21	4.05E+04 (8.09E+04)	3.57E+03 (7.72E+03)≈	2.61E+02 (1.50e+02)	1.13E+02 (1.16E+02)+	1.08E+02 (7.32E+01)	7.42E+01 (1.47E+01)+
F22	1.40E+02 (6.35E+01)	8.38E+01 (3.72E+01)+	8.90E+01 (6.05E+01)	7.13E+01 (4.16E+01)≈	2.49E+01 (2.15E+00)	2.53E+01 (3.47E+00)≈
F23	3.15E+02 (5.78E-14)	3.15E+02 (4.67E-14)≈	3.15E+02 (5.78E-14)	3.15E+02 (3.57E-14)≈	3.15E+02 (5.78e-14)	3.15E+02 (2.34e-13)≈
F24	2.25E+02 (2.62E+00)	2.23E+02 (2.16E+00)≈	2.25E+02 (2.65E+00)	2.24E+02 (2.10E+00)≈	2.25E+02 (2.73E+00)	2.21E+02 (2.14E+00)≈
F25	2.04E+02 (1.06E+00)	2.03E+02 (9.11E-01)≈	2.03E+02 (1.59E-01)	2.04E+02 (2.10E-01)−	2.03E+02 (1.00E-01)	2.03E+02 (1.05E-01)≈
F26	1.04E+02 (1.82E+01)	1.02E+02 (1.42E+01)≈	1.04E+02 (1.82E+01)	1.09E+02 (2.57E+01)≈	1.00E+02 (1.48E-02)	1.00E+02 (1.87E-02)≈
F27	3.24E+02 (4.34E+01)	3.38E+02 (5.24E+01)−	3.19E+02 (3.22E+01)	3.23E+02 (4.24E+01)≈	3.00E+02 (0.00E+00)	3.00E+02 (0.00E+00)≈
F28	7.82E+02 (5.39E+01)	7.98E+02 (4.97E+01)−	8.32E+02 (3.83E+01)	8.24E+02 (1.93E+01)≈	8.30E+02 (2.18E+01)	8.17E+02 (1.14E+01)+
F29	2.90E+05 (1.58E+06)	3.49E+03 (7.58E+03)+	7.23E+02 (8.18E+00)	7.14E+02 (7.16E+00)+	7.16E+02 (3.57E+00)	7.08E+02 (4.15E+00)+
F30	1.55E+03 (6.30E+02)	1.26E+03 (7.57E+02)≈	1.96E+03 (7.79E+02)	1.21E+03 (6.47E+02)+	1.25E+03 (6.07E+02)	8.67E+02 (7.17E+01)+
+		10		9		10
−		4		3		2
≈		16		18		18

**Table 3:** Results of DE algorithms with our scheme and original DE algorithms when function dimensionality is set 50. “+” denotes the result of a DE algorithm with our method is significant better than the result of its original DE algorithm in terms of Wilcoxon’s rank sum test at a 0.05 significance level, while “−” represents statistical worse. In addition, “≈” shows that there is no significant difference.

Function	Mean error (standard deviation)					
	JADE	JADE with our method	SHADE	SHADE with our method	L-SHADE	L-SHADE with our method
F1	1.54E+04 (1.01E+04)	3.42E+03 (5.27E+03)+	1.82E+04 (1.16E+04)	2.46E+03 (4.63E+02)+	7.29E+02 (1.58E+03)	2.46E+02 (1.67E+03)≈
F2	1.00E-13 (5.62E-14)	2.47E-15 (3.47E-15))≈	0.00E+00 (0.00E+00)	0.00E+00 (0.00E+00)≈	0.00E+00 (0.00E+00)	0.00E+00 (0.00E+00)≈
F3	4.23E+03 (1.98E+03)	7.48E+01 (2.74E+02)+	0.00E+00 (0.00E+00)	0.00E+00 (0.00E+00)≈	0.00E+00 (0.00E+00)	0.00E+00 (0.00E+00)≈
F4	2.78E+01 (4.40E+01)	2.57E+01 (7.16E+01)≈	3.01E+01 (4.54E+01)	2.88E+01 (6.89E+01)≈	4.00E+01 (4.65E+01)	2.14E+01 (2.77E+01)+
F5	2.04E+01 (4.03E-02)	2.01E+01 (2.27E-02)+	2.01E+01 (2.03E-02)	2.01E+01 (3.72E-02)≈	2.03E+01 (3.56E-02)	2.03E+01 (6.41E-02)≈
F6	1.62E+01 (6.59E+00)	1.51E+01 (4.21E+00)≈	3.01E+00 (1.48E+00)	4.25E+00 (2.48E+00)−	3.51E-01 (7.30E-01)	3.42E-01 (9.37E-01)≈
F7	6.57E-04 (2.50E-03)	4.13E-10 (3.69E-09)+	5.75E-04 (2.21E-03)	0.00E+00 (0.00E+00)+	0.00E+00 (0.00E+00)	0.00E+00 (0.00E+00)≈
F8	3.79E-15 (2.08E-14)	0.00E+00 (0.00E+00)≈	0.00E+00 (0.00E+00)	0.00E+00 (0.00E+00)≈	0.00E+00 (0.00E+00)	0.00E+00 (0.00E+00)≈
F9	5.19E+01 (7.18E+00)	3.86E+00 (6.73E+00)+	2.99E+01 (4.91E+00)	1.81E+01 (1.67E+00)+	1.14E+01 (1.88E+00)	1.57E+01 (3.48E+00)−
F10	6.20E-03 (7.87E-03)	1.97E-03 (5.62E-03)+	2.50E-03 (5.08E-03)	2.92E-03 (6.47E-03)−	4.34E-02 (2.46E-02)	1.82E-02 (3.69E-02)+
F11	3.82E+03 (4.06E+02)	3.34E+03 (8.72E+02)≈	3.46E+03 (2.72E+02)	1.73E+03 (4.72E+02)+	3.28E+03 (3.58E+02)	2.96E+03 (6.87E+02)≈
F12	2.60E-01 (3.77E-02)	1.74E-01 (2.79E-02)+	1.56E-01 (1.81E-02)	2.28E-01 (3.97E-02)−	2.23E-01 (2.77E-02)	1.97E-01 (6.46E-02)+
F13	3.18E-01 (4.39E-02)	2.83E-01 (5.67E-02)≈	3.13E-01 (3.94E-02)	1.88E-01 (1.39E-02)+	1.70E-01 (1.35E-02)	1.53E-01 (2.86E-02)+
F14	3.04E-01 (7.83E-02)	2.67E-01 (4.56E-02)+	3.13E-01 (9.90E-02)	3.27E-01 (1.87E-01)≈	3.10E-01 (2.07E-02)	3.13E-01 (3.47E-02)≈
F15	7.32E+00 (6.14E-01)	7.26E+00 (5.87E-01)≈	5.76E+00 (5.91E-01)	5.37E+00 (2.67E-01)+	5.11E+00 (4.37E-01)	4.87E+00 (9.46E-01)+
F16	1.77E+01 (4.61E-01)	1.25E+01 (3.16E-01)+	1.73E+01 (4.14E-01)	1.68E+01 (3.72E-01)≈	1.67E+01 (4.27E-01)	1.49E+01 (3.76E-01)≈
F17	2.38E+03 (5.90E+02)	2.44E+03 (3.73E+02)≈	2.53E+03 (6.12E+02)	2.14E+03 (7.34E+02)≈	1.34E+03 (3.53E+02)	1.53E+03 (6.72E+02)≈
F18	1.63E+02 (4.95E+01)	1.47E+02 (6.77E+01)≈	1.58E+02 (4.79E+01)	1.44E+02 (3.81E+01)≈	1.04E+02 (1.38E+01)	1.17E+02 (3.68E+01)≈
F19	1.18E+01 (4.05E+00)	8.83E+00 (7.42E+00)+	8.62E+00 (2.39E+00)	6.72E+00 (5.82E-01)+	8.42E+00 (2.05E+00)	6.27E+00 (3.47E+00)+
F20	7.40E+03 (6.14E+03)	1.37E+02 (3.69E+02)+	2.06E+02 (6.82E+01)	1.82E+02 (9.72E+01)≈	1.42E+01 (3.64E+00)	1.47E+01 (9.79E-01)≈
F21	1.39E+03 (4.13E+02)	1.27E+03 (7.46E+02)≈	1.36E+03 (3.04E+02)	8.91E+02 (7.69E+02)+	5.21E+02 (1.85E+02)	3.29E+02 (9.46E+01)+
F22	5.22E+02 (1.75E+02)	6.79E+02 (3.52E+02)−	3.90E+02 (1.39E+02)	4.12E+02 (4.72E+02)≈	1.02E+02 (6.50E+01)	9.81E+01 (4.38E+01)≈
F23	3.44E+02 (1.79E-13)	3.44E+02 (2.46E-13)≈	3.44E+02 (1.73E-13)	3.44E+02 (1.08E-13)≈	3.44E+02 (2.78E-13)	3.44E+02 (1.22E-13)≈
F24	2.74E+02 (2.37E+00)	2.79E+02 (3.68E+02)−	2.74E+02 (1.81E+00)	2.74E+02 (3.57E+00)≈	2.75E+02 (5.52E-01)	2.74E+02 (1.76E+00)≈
F25	2.16E+02 (6.55E+00)	2.15E+02 (5.43E+00)≈	2.07E+02 (3.97E+00)	2.05E+02 (6.71E+00)≈	2.05E+02 (3.46E-01)	2.08E+02 (1.79E-01)−
F26	1.07E+02 (2.53E+01)	1.09E+02 (3.46E+01)≈	1.00E+02 (1.18E-01)	1.02E+02 (1.03E-01)−	1.00E+02 (2.41E-02)	1.00E+02 (1.19E-02)≈
F27	4.60E+02 (5.18E+01)	4.71E+02 (6.34E+01)≈	4.16E+02 (4.75E+01)	4.23E+02 (3.98E+01)≈	3.40E+02 (3.68E+01)	3.51E+02 (2.67E+01)−
F28	1.12E+03 (5.96E+01)	1.29E+02 (7.59E+01)−	1.13E+03 (4.77E+01)	1.06E+03 (9.17E+00)≈	1.11E+03 (3.07E+01)	7.46E+02 (6.14E+00)+
F29	8.77E+02 (5.47E+01)	8.65E+02 (6.49E+01)≈	8.96E+02 (8.27E+01)	6.97E+02 (9.37E+00)+	8.13E+02 (4.79E+01)	6.42E+02 (3.72E+01)+
F30	9.84E+03 (1.01E+03)	9.47E+03 (5.35E+02)≈	9.42E+03 (7.72E+02)	8.16E+03 (5.71E+02)+	8.81E+03 (4.22E+02)	7.36E+03 (3.43E+02)+
+		11		10		9
−		3		4		3
≈		16		16		18

**Table 4:** Results of DE algorithms with our scheme and original DE algorithms when function dimensionality is set 100. “+” denotes the result of a DE algorithm with our method is significant better than the result of its original DE algorithm in terms of Wilcoxon’s rank sum test at a 0.05 significance level, while “−” represents statistical worse. In addition, “≈” shows that there is no significant difference.

Function	Mean error (standard deviation)					
	JADE	JADE with our method	SHADE	SHADE with our method	L-SHADE	L-SHADE with our method
F1	1.01E+05 (5.57E+04)	7.01E+05 (6.75E+04)+	1.47E+05 (7.31E+04)	8.24E+04 (4.25E+04)+	1.51E+05 (4.70E+04)	9.88E+04 (7.64E+03)+
F2	6.79E-10 (1.20E-09)	5.46E-12 (3.87E-10)≈	0.00E+00 (0.00E+00)	0.00E+00 (0.00E+00)≈	0.00E+00 (0.00E+00)	0.00E+00 (0.00E+00)≈
F3	5.91E+03 (3.00E+03)	1.46E+02 (8.45E+02)≈	4.70E-03 (8.84E-03)	5.72E-03 (4.71E-03)≈	0.00E+00 (0.00E+00)	0.00E+00 (0.00E+00)≈
F4	8.64E+01 (6.21E+01)	8.45E+01 (3.79E+01)≈	1.18E+02 (5.47E+01)	8.87E+01 (7.94E+01)+	1.72E+02 (3.18E+01)	1.29E+02 (2.76E+01)+
F5	2.05E+01 (3.40E-02)	2.04E+01 (6.72E-02)≈	2.02E+01 (1.57E-02)	2.00E+01 (1.94E-02)+	2.05E+01 (4.08E-02)	2.06E+01 (5.67E-02)−
F6	4.55E+01 (1.60E+01)	2.75E+01 (8.64E+00)+	2.99E+01 (4.78E+00)	2.37E+01 (6.72E+00)≈	9.32E+00 (1.95E+00)	6.72E+00 (3.48E+00)+
F7	1.07E-03 (2.80E-03)	1.24E-03 (3.49E-03)≈	1.72E-03 (4.10E-03)	0.00E+00 (0.00E+00)≈	0.00E+00 (0.00E+00)	0.00E+00 (0.00E+00)≈
F8	1.14E-13 (0.00E+00)	0.00E+00 (0.00E+00)≈	0.00E+00 (0.00E+00)	0.00E+00 (0.00E+00)≈	1.20E-03 (6.93E-04)	1.25E-13 (7.00E-12)−
F9	1.47E+02 (2.02E+01)	6.94E+01 (8.46E+00)+	9.82E+01 (1.42E+01)	5.72E+01 (9.72E+00)+	3.69E+01 (4.82E+00)	2.72E+01 (1.72E+00)+
F10	1.35E-02 (9.14E-03)	8.43E-02 (6.37E-03)+	5.62E-03 (4.74E-03)	3.72E-03 (6.79E-03)+	1.71E+01 (4.01E+00)	1.43E+01 (5.78E-01)≈
F11	1.05E+04 (6.04E+02)	9.47E+03 (7.46E+02)≈	9.80E+03 (6.38E+02)	6.72E+03 (3.72E+02)+	1.08E+04 (4.58E+02)	9.27E+03 (7.71E+02)≈
F12	3.42E-01 (2.66E-02)	3.57E-01 (4.19E-02)−	2.30E-01 (2.26E-02)	3.31E-01 (7.49E-02)−	4.13E-01 (4.29E-02)	3.76E-01 (7.50E-02)≈
F13	4.05E-01 (5.01E-02)	3.99E-01 (6.74E-02)≈	4.10E-01 (4.19E-02)	4.16E-01 (3.71E-02)≈	2.41E-01 (1.85E-02)	1.71E-01 (2.71E-02)+
F14	3.18E-01 (2.77E-02)	2.48E-01 (1.73E-02)+	2.09E-01 (1.55E-02)	2.07E-01 (2.43E-02)≈	2.24E-01 (1.40E-02)	2.18E-01 (2.48E-02)≈
F15	2.90E+01 (3.55E+00)	2.87E+01 (2.87E+00)≈	1.93E+01 (1.87E+00)	1.88E+01 (2.57E+00)≈	1.57E+01 (1.00E+00)	1.64E+01 (3.64E+00)≈
F16	4.00E+01 (4.06E-01)	4.02E+01 (4.87E-01)−	3.97E+01 (5.65E-01)	3.95E+01 (4.74E-01)≈	3.92E+01 (4.74E-01)	3.84E+01 (3.48E-01)≈
F17	1.27E+04 (6.21E+03)	1.11E+04 (9.47E+03)≈	1.09E+04 (4.71E+03)	6.70E+03 (2.56E+03)+	4.47E+03 (7.75E+02)	3.82E+03 (9.71E+02)≈
F18	9.34E+02 (1.03E+03)	7.56E+02 (3.48E+03)≈	7.94E+02 (5.08E+02)	5.48E+02 (6.79E+02)+	2.17E+02 (1.30E+01)	1.94E+02 (2.78E+01)≈
F19	9.47E+01 (1.99E+01)	9.32E+01 (1.79E+01)+	9.82E+01 (1.11E+01)	9.64E+01 (9.65E+00)+	9.62E+01 (2.42E+00)	9.34E+01 (3.57E+00)+
F20	9.63E+03 (1.54E+04)	6.36E+03 (3.71E+03)+	5.92E+02 (1.45E+02)	4.87E+02 (1.78E+02)≈	1.52E+02 (4.21E+01)	1.48E+02 (6.45E+01)≈
F21	3.79E+03 (1.03E+03)	3.49E+03 (8.47E+02)≈	3.36E+03 (1.07E+03)	3.10E+03 (2.87E+03)≈	2.21E+03 (5.20E+02)	2.34E+03 (9.45E+02)≈
F22	1.61E+03 (2.62E+02)	1.58E+03 (3.94E+02)≈	1.36E+03 (2.83E+02)	1.58E+03 (1.72E+02)−	1.03E+03 (1.83E+02)	1.12E+03 (4.57E+02)≈
F23	3.48E+02 (9.52E-13)	3.48E+02 (7.67E-13)≈	3.48E+02 (9.61E-13)	3.48E+02 (5.18E-13)≈	3.48E+02 (1.89E-13)	3.48E+02 (5.64E-13)≈
F24	3.99E+02 (5.39E+00)	4.01E+02 (6.72E+00)≈	3.97E+02 (4.23E+00)	3.95E+02 (6.25E+00)≈	3.95E+02 (2.83E+00)	3.95E+02 (1.87E+00)≈
F25	2.73E+02 (4.87E+00)	2.83E+02 (7.19E+00)≈	2.64E+02 (5.19E+00)	2.31E+02 (6.71E+00)+	2.00E+02 (2.60E-13)	2.00E+02 (1.92E-13)≈
F26	2.00E+02 (4.68E-03)	2.01E+02 (8.64E-03)≈	2.00E+02 (5.86E-03)	2.00E+02 (7.37E-03)≈	2.00E+02 (2.38E-13)	2.00E+02 (6.24E-03)≈
F27	1.08E+03 (1.23E+02)	8.27E+02 (9.72E+01)+	8.94E+02 (1.03E+02)	6.76E+02 (9.64E+02)≈	3.80E+02 (3.28E+01)	2.54E+02 (6.72E+01)+
F28	2.38E+03 (2.65E+02)	2.41E+03 (3.72E+02)≈	2.45E+03 (2.94E+02)	2.37E+03 (1.73E+02)≈	2.24E+03 (4.61E+01)	2.01E+03 (3.72E+01)+
F29	1.36E+03 (1.72E+02)	9.09E+02 (1.43E+02)+	1.23E+03 (2.62E+02)	8.45E+02 (3.81E+02)+	7.69E+02 (5.20E+01)	4.67E+02 (2.67E+02)+
F30	8.60E+03 (1.35E+03)	8.34E+03 (8.74E+02)≈	8.76E+03 (9.51E+02)	8.81E+03 (7.62E+02)≈	8.30E+03 (6.56E+02)	8.18E+03 (5.48E+02)≈
+		9		11		9
−		2		2		2
≈		19		17		19

**Fig 1:**
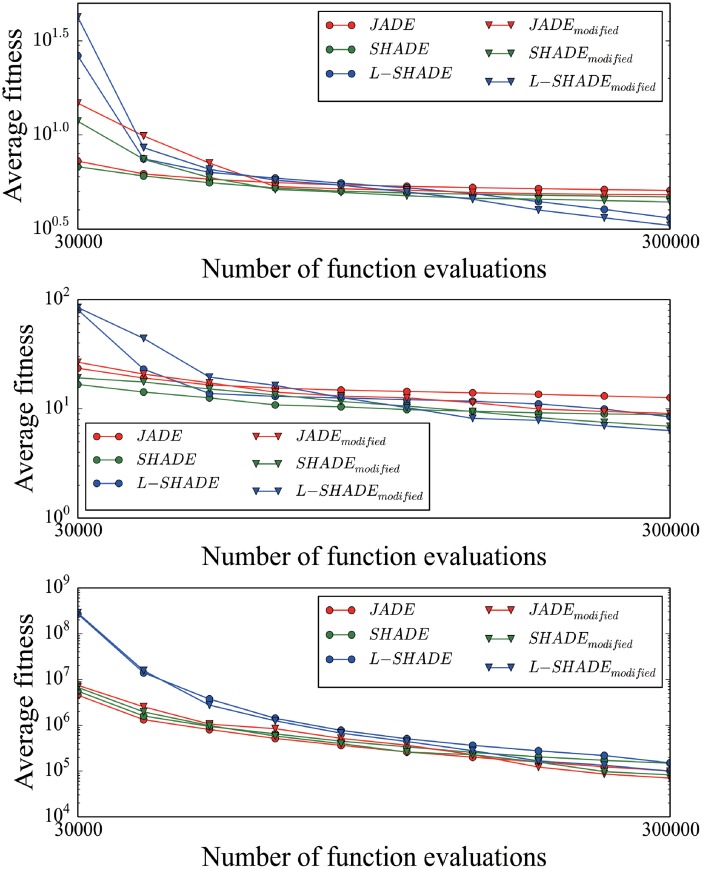
Convergence graphics. A: The convergence graphic for F19 when function dimensionality is 30. B: The convergence graphic for F19 when function dimensionality is 50. C: The convergence graphic for F1 when function dimensionality is 100.

It can be seen from Tables 2–4 that, for some functions, all DE algorithms with our method significantly win their original DE algorithms. Details go as below. When function dimensionality is 30, for F19 and F29, our method lead to significant improvement in all the cases. When function dimensionality goes to 50, for F19, our method lead to significant improvement in all the cases. When function dimensionality becomes 100, for F1, F9, F19, and F29, our method lead to significant improvement in all the cases. Based on the mean error to the optimum at interval, we plot convergence graphics of runs for one function when function dimensionality is 30, 50, and 100, respectively, in Fig 1.

As shown in Fig 1, convergence rate goes lower and lower in all runs. In the figure, runs with our scheme converge more slowly at the initial stage than runs without it but faster at the remaining part. The above phenomenon can be explained as below. The original value of population size in the DE algorithms *NP*_*o*_ has been proven to be a fitting value by experiments in literate. In theory, size of each subpopulation in the DE algorithms with our method need be set *NP*_*o*_. That is, population size need be set 2 ⋅ *NP*_*o*_. However, due to the limitation in maximum fitness evaluations, great increase of population size means great decrease of maximum generations. Therefore, subpopulation size needs be set less than *NP*_*o*_ to ensure that enough generations can be executed in runs. At the beginning of run, DE algorithms with our scheme converge more slowly than original DE algorithms for the lack of individuals in each subpopulation. Nevertheless, with the implement of our scheme, the disadvantage is offset gradually in many cases. Altogether, our method leads to significant improvement in 88 cases out of 270 ones but statistical deterioration in 25 cases. In summary, JADE and its variants with our scheme outperform their original version.

### Comparison between L-SHADE with our scheme and up-to-date DE algorithms

According to Tables 2–4, L-SHADE based on our scheme is best in performance among the three DE algorithm with our scheme. Thus, we plan to compare L-SHADE based on our scheme with up-to-date DE algorithms, CoBiDE, MPEDE, EDEV, and MLCCDE. In Table 5, the experimental results are listed.

**Table 5:** Results of L-SHADE based on our method, MLCCDE, EDEV, MPEDE and CoBiDE when function dimensionality is set 30. “+” denotes that the result of L-SHADE based on our method is significant better than the current result in terms of Wilcoxon’s rank sum test at a 0.05 significance level, while “−” represents statistical worse. Meanwhile, “≈” shows that there is no significant difference.

Function	Mean error (standard deviation)				
	L-SHADE based on our method	MLCCDE	EDEV	MPEDE	CoBiDE
F1	0.00E+00 (0.00E+00)	7.20E+03 (5.39E+03)+	1.88E+03 (5.74E+03)+	9.43E-11 (4.77E-10)≈	1.46E+04 (1.05E+04)+
F2	0.00E+00 (0.00E+00)	0.00E+00 (0.00E+00)≈	0.00E+00 (0.00E+00)≈	9.47E-16 (5.19E-15)≈	0.00E+00 (0.00E+00)≈
F3	0.00E+00 (0.00E+00)	0.00E+00 (0.00E+00)≈	0.00E+00 (0.00E+00)≈	4.36E-14 (2.45E-14)≈	0.00E+00 (0.00E+00)≈
F4	0.00E+00 (0.00E+00)	0.00E+00 (0.00E+00)≈	2.23E+01 (8.06E+01)≈	6.37E-08 (3.48E-07)≈	2.66E-06 (8.45E-06)≈
F5	2.01E+01 (2.24E-02)	2.02E+01 (4.73E-02)≈	2.04E+01 (6.21E-02)+	2.04E+01 (4.98E-02)≈	2.04E+01 (2.48E-01)+
F6	0.00E+00 (0.00E+00)	2.99E-02 (1.63E-01)≈	6.23E-01 (9.03E-01)≈	9.57E-01 (9.78E-01)≈	1.23E+00 (1.23E+00)+
F7	0.00E+00 (0.00E+00)	0.00E+00 (0.00E+00)≈	0.00E+00 (0.00E+00)≈	5.75E-04 (2.21E-03)≈	0.00E+00 (0.00E+00)≈
F8	0.00E+00 (0.00E+00)	0.00E+00 (0.00E+00)≈	0.00E+00 (0.00E+00)≈	1.52E-14 (3.93E-14)≈	0.00E+00 (0.00E+00)≈
F9	4.21E+00 (9.72E-01)	2.29E+01 (4.03E+00)+	3.27E+01 (4.97E+00)+	2.81E+01 (5.90E+00)+	4.14E+01 (1.07E+01)+
F10	1.39E-03 (5.28E-03)	2.82E-01 (3.45E-01)+	6.27E-03 (9.69E-03)≈	1.14E+00 (5.10E-01)+	5.90E+01 (1.39E+01)+
F11	1.03E+03 (1.16E+02)	1.82E+03 (2.76E+02)+	2.47E+03 (5.39E+02)+	2.41E+03 (3.42E+02)+	1.65E+03 (4.48E+02)≈
F12	1.48E-01 (2.48E-02)	2.32E-01 (6.46E-02)≈	6.13E-01 (1.42E-01)+	5.07E-01 (9.11E-02)+	2.45E-01 (3.48E-01)≈
F13	1.14E-01 (1.13E-02)	1.82E-01 (2.61E-02)≈	1.94E-01 (3.01E-02)≈	2.15E-01 (3.68E-02)+	2.36E-01 (4.76E-02)+
F14	2.09E-01 (1.48E-02)	1.98E-01 (2.36E-02)≈	1.83E-01 (2.97E-02)≈	2.42E-01 (3.60E-02)≈	2.23E-01 (3.53E-02)≈
F15	2.02E+00 (4.52E-01)	2.35E+00 (5.93E-01)≈	4.13E+00 (5.69E-01)+	4.14E+00 (7.37E-01)+	3.10E+00 (8.50E-01)≈
F16	8.58E+00 (3.47E-01)	9.10E+00 (5.46E-01)≈	9.84E+00 (3.79E-01)≈	1.00E+01 (4.93E-01)+	9.94E+00 (6.90E-01)+
F17	1.49E+02 (8.41E+01)	3.19E+02 (1.81E+02)+	2.22E+03 (4.85E+03)+	2.26E+02 (1.61E+02)+	2.26E+02 (1.80E+02)+
F18	6.64E+00 (1.49E+00)	1.63E+01 (5.94E+00)+	3.23E+01 (2.01E+01)+	1.35E+01 (5.69E+00)+	1.06E+01 (3.75E+00)+
F19	3.11E+00 (6.74E-01)	2.57E+00 (5.73E-01)−	4.30E+00 (2.37E+00)+	3.87E+00 (6.68E-01)+	2.65E+00 (4.39E-01)≈
F20	2.25E+00 (2.74E+00)	9.33E+00 (5.58E+00)+	1.52E+01 (3.27E+00)+	9.61E+00 (2.83E+00)+	7.30E+00 (2.73E+00)+
F21	7.42E+01 (1.47E+01)	1.32E+02 (9.70E+01)+	4.07E+02 (3.22E+02)+	1.32E+02 (9.84E+01)+	1.08E+02 (9.88E+01)≈
F22	2.53E+01 (3.47E+00)	5.73E+01 (5.72E+01)+	1.13E+02 (5.55E+01)+	9.07E+01 (6.36E+01)+	1.07E+02 (7.26E+01)+
F23	3.15E+02 (2.34e-13)	3.15E+02 (5.78E-14)≈	3.14E+02 (1.97E-13)≈	3.15E+02 (5.78E-14)≈	3.15E+02 (5.78E-14)≈
F24	2.21E+02 (2.14E+00)	2.24E+02 (8.82E-01)≈	2.24E+02 (8.55E-01)≈	2.25E+02 (1.46E+00)≈	2.22E+02 (4.25E+00)≈
F25	2.03E+02 (1.05E-01)	2.03E+02 (4.86E-01)≈	2.01E+02 (2.89E+00)≈	2.00E+02 (2.24E-03)≈	2.03E+02 (3.80E-01)≈
F26	1.00E+02 (1.87E-02)	1.00E+02 (2.08E-02)≈	1.04E+02 (1.82E+01)+	1.00E+02 (2.70E-02)≈	1.00E+02 (5.92E-02)≈
F27	3.00E+02 (0.00E+00)	3.32E+02 (4.68E+01)≈	3.61E+02 (4.93E+01)≈	3.59E+02 (4.90E+01)≈	3.93E+02 (2.38E+01)≈
F28	8.17E+02 (1.14E+01)	8.01E+02 (2.62E+01)≈	3.83E+02 (7.76E+00)−	8.34E+02 (3.35E+01)≈	8.20E+02 (2.82E+01)≈
F29	7.08E+02 (4.15E+00)	7.06E+02 (1.04E+02)≈	2.14E+02 (9.47E-01)−	2.98E+05 (1.63E+06)+	5.69E+02 (2.48E+02)≈
F30	8.67E+02 (7.17E+01)	6.08E+02 (2.28E+02)−	3.49E+02 (1.11E+02)−	6.69E+02 (1.69E+02)≈	7.05E+02 (2.83E+02)≈
+		9	13	14	11
−		2	3	0	0
≈		19	14	16	19

It can be seen from the table that L-SHADE based on our method significantly wins MLCCDE, EDEV, MPEDE and CoBiDE in 9, 13, 14 and 11 cases, respectively. Meanwhile, L-SHADE based on our method loses to MLCCDE, EDEV, MPEDE and CoBiDE in two, three, zero and zero cases, respectively. There is no significant difference in all of other cases. In summary, L-SHADE with our method is very competitive.

### Discussion

In JADE and its variants with our scheme, the two subpopulations evolve independently. Individuals in a subpopulation, compared with individuals in the other subpopulation, are different in chromosome but similar in fitness level. Therefore, regarding the other subpopulation as the optional external archive can provide fitting individuals for difference operation. Under the control of our scheme, DE/current-to-*p*best/1 with optional archive become more efficient than before. Thus, DE algorithms based on DE/current-to-*p*best/1 with optional archive become more powerful than before. In fact, if maximum fitness evaluations can be extended, DE algorithms with our scheme may have a more significant advantage.

## Conclusion

JADE and its variants, DE algorithms based on DE/current-to-*p*best/1 with optional archive, show good performance in comparisons among DE algorithms. Nevertheless, more powerful DE algorithms are needed for the solving difficulty arisen from the complexity of problems. The mutation strategy of these DE algorithms, DE/current-to-*p*best/1 with optional archive, is based on the external optional archive. In this paper, we propose a new scheme for managing the archive. According to our scheme, two subpopulations are maintained in the population. Each of them regards the other as its archive. In this way, the individuals in the archive of a subpopulation are ones similar in fitness level with current target vectors but different in building blocks of chromosome from current target vectors. Experiments based on the CEC2014 benchmark functions not only show that our scheme can significantly improve solutions of JADE and its two variants, SHADE and L-SHADE, but also demonstrate that L-SHADE with our method performs significantly better than CoBiDE, MPEDE, EDEV, and MLCCDE.

As mentioned above, our scheme for managing the archive is enlightened by DEA. Conversely, a new type of distributed DE—DEA in the field of DE—can be developed based on the work in this paper. Further investigation is remained to be done.
